# Integrated *in vivo* and *in silico* validation of *Corchorus olitorius* seed-mediated cardioprotection against doxorubicin-induced cardiotoxicity via network pharmacology, molecular docking, and molecular dynamics

**DOI:** 10.3389/fchem.2026.1810825

**Published:** 2026-07-30

**Authors:** Fatma Mohamed Abd El-Mordy, Sherif A. Maher, Omar Y. Tammam, Mahmoud A. Elrehany, Mohamed Hisham, Remon R. Rofaeil, Mostafa E. Rateb, Hesham F. Oraby, Mahmoud A. A. Ibrahim, Ahmed M. Shawky, Gerhard Bringmann, Usama Ramadan Abdelmohsen, Nourhan Hisham Shady

**Affiliations:** 1 Department of Pharmacognosy and Medicinal Plants, Faculty of Pharmacy (Girls), Al-Azhar University, Cairo, Egypt; 2 Department of Biochemistry, Faculty of Pharmacy, New Valley University, El-kharga, Egypt; 3 Department of Biochemistry, Faculty of Medicine, Minia University, Minia, Egypt; 4 Department of Biochemistry, Faculty of Pharmacy, Deraya University, Universities Zone, New Minia City, Egypt; 5 Department of Pharmaceutical Chemistry, Faculty of Pharmacy, Deraya University, New Minia, Egypt; 6 Department of Pharmacology, Deraya University, Universities Zone, New Minia City, Egypt; 7 Natural and Medical Sciences Research Center, University of Nizwa, Nizwa, Oman; 8 Biotechnology/Molecular Biology and Biomedical Sciences Programs, Faculty of Basic and Applied Science, Egypt-Japan University of Science and Technology (EJUST), New Borg El-Arab City, Alexandria, Egypt; 9 School of Health Sciences, University of KwaZulu-Natal, Durban, South Africa; 10 Department of Supportive Requirements, University of Technology and Applied Sciences, Nizwa, Oman; 11 Department of Chemistry, Faculty of Science, Umm Al-Qura University, Makkah, Saudi Arabia; 12 Institute of Organic Chemistry, University of Würzburg, Am Hubland, Würzburg, Germany; 13 Department of Pharmacognosy, Faculty of Pharmacy, Minia University, Minia, Egypt; 14 Department of Pharmacognosy, Faculty of Pharmacy, Deraya University, Universities Zone, New Minia City, Egypt; 15 Center for Entrepreneurship and Research in Sustainability, Deraya University, Universities Zone, New Minia City, Egypt

**Keywords:** AKT1, cardiac glycosides, cardioprotection, *Corchorus olitorius*, doxorubicin-induced cardiotoxicity, molecular docking, network pharmacology, NF-κB

## Abstract

**Background:**

*Corchorus olitorius* L., known in Arab countries as molokhia, is a leafy plant rich in many carotenoids, minerals, and vitamins; its seeds are also reported to offer cardioprotective effects. Although the cardioprotective effects of molokhia are ascribed to the presence of cardiac glycoside compounds, the exact action mechanisms remain uninvestigated.

**Methods:**

In this study, we examined the potential of molokhia seeds to protect against cardiopathy by isolating the major constituents of *C. olitorius* seeds, analyzing its gene expression *in vivo,* and conducting computational studies to demonstrate the cardioprotective activities of these constituents.

**Results:**

Crude extracts of *C. olitorius* seeds were found to reduce the expression of *IL-1β*, *IL-6*, *TNF-α*, *NF-κB*, *ICAM-1*, and *MMP-9* that had increased upon the administration of doxorubicin (DOX). Electrocardiographic studies showed reduced heart rate along with prolongation of the PR interval, QRS complex, and ST segment upon *Corchorus* administration.

**Conclusion:**

The findings of this study demonstrate that the cardioprotective effects of molokhia seeds against DOX-induced cardiotoxicity are probably mediated via anti-inflammatory actions.

## 1 Introduction

*Corchorus olitorius* L. (also known as molokhia) is a green leafy vegetable (Manvi and Khan, 2026) belonging to the family Tiliaceae (Helaly et al., 2017) and is widely consumed in African and Middle Eastern cultures as a thick soup. This plant has good nutritional value owing to its high content of carotenoids, minerals, and vitamins A, B, C, and E (Ahmed, 2021). A literature review on *C. olitorius* shows that it has several medicinal purposes (Abdel-Razek et al., 2022); the seeds of this plant are known to have mild laxative, demulcent, diuretic, and estrogenic activities (Shady et al., 2022). However, one report noted that cattle died after accidentally consuming these seeds in their meal, which was attributed to the presence of appreciable amounts of cardiac glycosides in the seeds (Matsufuji et al., 2001).

Cardiac glycosides are steroids that act specifically and strongly on the heart muscle (Jiao et al., 2026); hence, a very small amount of these compounds can significantly stimulate a diseased heart. These substances are most useful for treating congestive heart failure (CHF) as they can strengthen the contractions of the heart without increasing oxygen consumption. As a result, the myocardium can meet the needs of the circulatory system and become a more effective pump (Morsy, 2017). Phytochemical investigations showed that cardiac glycosides are the major constituents of *C. olitorius* seeds and that these are important pharmaceuticals that are widely used in the treatment of heart failure. Strophanthidin glycosides are the most common type of cardiac glycosides present in the *Corchorus* species of plants and include coroloside, erysimoside, olitoriside, corchorosides A and B, veticoside, helveticoside, and Corchorusoides A-E (Al-Yousef et al., 2017; Matsufuji et al., 2001).

Doxorubicin (DOX)-induced cardiac failure involves many processes. DOX-induced cardiomyopathy is significantly linked to elevated levels of myocardial oxidative stress, as evidenced by the damage caused by reactive oxygen species (ROS), including lipid peroxidation and reduced levels of antioxidants and sulfhydryl groups. Moreover, myofibrillar degeneration and intracellular calcium dysregulation are significant pathways related to DOX-induced heart damage. Caspase activation and internucleosomal DNA degradation indicate that DOX-induced apoptosis impacts both cardiomyocytes and endothelial cells (Octavia et al., 2012).

In this study, we describe the separation and structural elucidation of eight metabolites from two different extracts of *C. olitorius* seeds. The isolated compounds include five cardenolide glycosides, two steroidal compounds, and a trisaccharide. Furthermore, we proved the effects of a crude extract of *C. olitorius* seeds on the relative expression of *IL-1β*, *IL-6*, *TNF-α*, *NF-ΚB*, *ICAM-1*, and *MMP-9* in the cardiac tissues of rats treated with DOX. Finally, we conducted computational studies using network-pharmacology-based analysis and molecular docking to comprehend the mechanisms and pathways by which the compounds isolated from *C. olitorius* seeds act in the treatment of CHF. We employed network pharmacology, molecular docking, and molecular dynamics (MD) analyses as mechanistic support tools rather than stand-alone discovery platforms to interpret and validate the experimentally observed cardioprotective effects.

## 2 Materials and methods

### 2.1 Plant material

Seeds of *C. olitorius* were collected in February 2021 from Minia Governorate, Egypt. The botanical authentication was carried out by Dr. Abd El-Halim A. Mohammed (Horticultural Research Institute, Flora and Phyto-taxonomy Research Department, Cairo, Egypt). A reference voucher specimen (Corc-2-2021) for this plant has been deposited in the Department of Pharmacognosy, Faculty of Pharmacy, Deraya University, Egypt.

### 2.2 *C. olitorius* seed extract preparation

An extract was exhaustively prepared from the dried seeds of *C. olitorius* (2 kg) by first macerating with 70% aqueous methanol (5 L per cycle, four successive extractions for 3rd day each) at room temperature (20 °C). The combined extracts were then filtered and concentrated under reduced pressure at 40 °C using a rotary evaporator (Büchi Rotavapor R-300, Vernon Hills, United States) to yield 200 g of the crude extract. This extract was resuspended in distilled water (0.5 L) and subjected to liquid–liquid partitioning using solvents of ascending polarity. Fractionation was performed successively with dichloromethane (4 × 1 L), ethyl acetate (3 × 1 L), *n*-butanol (3 × 1 L), and methanol (4 × 1 L). After each extraction step, the organic layer was separated and evaporated under vacuum at 45 °C, which yielded Fraction I (68.0 g), Fraction II (3.0 g), Fraction III (7.0 g), and Fraction IV (82.0 g) correspondingly. All obtained fractions were preserved at 4 °C for both phytochemical and biological studies. Since Fractions I and IV were heavier and showed more promising thin-layer chromatography (TLC) spotting than Fractions II and III, we decided to focus on the heavier fractions in this work.

### 2.3 Isolating the major constituents of *C. olitorius* seeds

Fraction I (68.0 g) was subjected to normal vacuum liquid chromatography (VLC) using dichloromethane (DCM) to methanol (MeOH) successive elution (0%–100%, 5% increase, 100 mL each). Similar fractions were gathered and vacuum-evaporated to obtain two major subfractions *M*_*1*_ and *M*_*2*_. Subfraction *M*_*1*_ (20.0 g) was extensively fractionated on another normal silica VLC column; here, gradient chromatographic elution was carried out using *n-*hexane and ethyl acetate (0%–100%, 5% increments, 100 mL each) to afford three subfractions *I*_*1*_ *–I*_*3*_ by collecting similar fractions together and performing vacuum evaporation. Next, subfractions *I*_*1*_ (0.5 g) and *I*_*2*_ (1.0 g) were each slightly chromatographed on a Sephadex™ LH-20 column eluted with MeOH to afford compound 1 (9 mg); subfraction *I*_*3*_ (15.0 g) was separated using silica gel 60 (100 × 1 cm, 20 g). Chromatographic elution was carried out using petroleum ether to ethyl acetate gradient elution (0%–100%, 5% increments, 200 mL each) to yield compound 2 (5 mg) and a collective fraction, which was further chromatographed on a reversed-phase silica VLC column using water (H_2_O) and MeOH (0%–100%, 5% increments, 100 mL each) to obtain compound 3 (30 mg) and compound 4 (5 mg).

Fraction IV (82.0 g) was suspended in 10% MeOH in H_2_O and separated on a polyamide S (Fluka, St. Louis, MO, United States) column (150 g, 100 × 3 cm) using gradient elution with distilled water (H_2_O/MeOH, 0%–100%, 10% increments, 500 mL each) to obtain 11 subfractions (*P*_*1*_ *–P*_*11*_). Here, subfraction *P*_*3*_ (5.0 g) (100% water) was chromatographed on a Sephadex™ LH-20 column using an isocratic mixture of H_2_O/MeOH (30%, 500 mL) to obtain compound 5 (30 mg); next, subfraction *P*_*4*_ (7.0 g; eluted with 10%–20% MeOH and water) was subjected to reverse-phased VLC using H_2_O and MeOH (0%–100%, 10% increments, 100 mL each) to obtain nine subfractions (1–9), of which the ninth subfraction was slightly chromatographed on a Sephadex™ LH-20 column using an isocratic mixture of H_2_O/MeOH (30%, 300 mL) to obtain compound 6 (40 mg). Finally, subfraction *P*_*5*_ (9.0 g; eluted with 20%–30% MeOH and water) was chromatographed on a normal silica VLC column; successive elution with DCM to MeOH (0%–100%, 10% increments, 100 mL each) yielded 11 subfractions (I–XI), among which subfraction IV and collective subfractions V–X were further chromatographed on a normal silica VLC column using DCM/MeOH (0%–100%, 10% increments, 100 mL each) to obtain compound 7 (6 mg) and compound 8 (3 mg), respectively.

### 2.4 Experimental animals

Adult male Wistar albino rats (200–240 g) were obtained from the animal facility of the Faculty of Pharmacy, Deraya University, Minia, Egypt. Prior to the experiments, the animals were housed under controlled environmental conditions and allowed a 7-day acclimation period with 12/12 h light/dark cycles at a regulated temperature and free access to standard laboratory chow and drinking water.

#### 2.4.1 Ethics statement

Scarification, medication, and animal handling were performed in accordance with the NIH Guide for Care and Use of Laboratory Animals as well as the guidelines for care of experimental animals provided by the Institutional Ethical Committee of Deraya Center for Scientific Research, Deraya University, Minia, Egypt. The animal study was approved by the Ethics Committee of the Faculty of Pharmacy of Deraya University, which also declared that all testing should be conducted in accordance with the Guide for the Care and Use of Laboratory Animals and that no animal should be subjected to pain (ethical permission no. N10/2021).

No human studies are presented in this work. The study was conducted in accordance with all local legislation and institutional requirements. No potentially identifiable images or data are presented in this manuscript.

#### 2.4.2 Experimental design

The experimental animals (21 rats) were divided into three groups of seven rats each and numbered as Groups I to III as follows:

*Group I:* Normal control group comprising rats that orally receive 0.9% saline solution once daily for 1 week (7 days).

*Group II:* DOX-treated group receiving 0.9% saline solution orally once daily for the first 6 days as pretreatment. On day 7, the rats were given a single intraperitoneal dose of DOX (2 mg/kg) (Jacquet et al., 1997; Zhang et al., 2005).

*Group III: C. olitorius* seed crude extract treatment group receiving the extract dissolved in propylene glycol (PG; 25 mg/kg bodyweight) once daily for 6 days as pretreatment. On day 7, the rats received a single intraperitoneal dose of DOX (2 mg/kg).

After experiment completion, the rats were anesthetized by urethane (1 g/kg intraperitoneally); the cardiac tissue was then dissected, cleansed with 1× phosphate-buffered saline (PBS, pH 7.4, with a final concentration of 10 mM PO_4_^3–^, 137 mM NaCl, and 2.7 mM KCl) in ice, and dried and weighed in a 1:5 ratio of tissue weight to homogenization buffer. The cardiac tissue samples were maintained in RNAlater and preserved at 4 °C for the first 24 h before storing at −20 °C for measuring the mRNA components via quantitative reverse transcription polymerase chain reaction (qRT-PCR).

##### 2.4.2.1 Assessment of cardiac function by surface electrocardiogram (ECG)

The rats were given a mild anesthesia using 1.5% isoflurane in oxygen. Then, they were warmed on a heating pad at 37 °C in a supine position. To record the ECG, the limbs of the rats were connected to an MP160 polygraph machine (BIOPAC, Goleta, CA, United States).

##### 2.4.2.2 Safety considerations

Safety considerations are critical when evaluating the extract of *C. olitorius* seeds given the known cardiac glycoside content. Previous toxicological studies have reported that ethanolic seed extracts exhibit an oral LD_50_ greater than 5,000 mg/kg in rats, with no mortality observed at doses up to 1,250 mg/kg/day for 28 days, although biochemical and histological alterations were noted at higher chronic doses (2,500–3,750 mg/kg/d) (Egua et al., 2014). Similarly, earlier toxicological investigations confirmed that the seeds were among the most glycoside-rich parts of the plant and could be lethal to small animals at very high doses when administered intraperitoneally (Negm et al., 1980). By contrast, the dose employed in our study (25 mg/kg/day orally for 6 days) was markedly lower by more than two orders of magnitude below the reported LD_50_ values and well beneath doses associated with biochemical toxicity. Importantly, no overt adverse effects were observed in our acute model.

### 2.5 Gene expression analysis

#### 2.5.1 Total RNA extraction

Total RNA was isolated from the cardiac tissue using TRIzol™ extraction reagent (Invitrogen, United States) in accordance with the manufacturer protocols. Accordingly, approximately 100 mg of the cardiac tissue was homogenized in 1 mL of TRIzol™ using an ultrasonic homogenizer (Vibracell, Sonics and Materials Inc., Newtown, United States) prior to RNA separation (Hummon et al., 2007). The concentration of the extracted RNA was quantified by measuring the absorbance at 260 nm, and the sample purity was assessed using the A260/A280 ratio. Only RNA samples with purity values ≥1.7 were selected for subsequent qRT-PCR analyses and relative gene expression studies.

#### 2.5.2 Real-time qRT-PCR

cDNA was synthesized from equal amounts of total RNA from each sample using the RevertAid H Minus First Strand cDNA Synthesis Kit (catalog no. K1632, Thermo Scientific Fermentas, St. Leon-Rot, Germany) as per manufacturer protocols. Then, real-time PCR was performed using the resulting single-stranded cDNA templates, and amplification reactions were carried out using the Maxima SYBR Green qPCR Master Mix (catalog no. K0251, Thermo Scientific Fermentas) on a StepOne real-time PCR detection system (Applied Biosystems). Gene-specific primers were designed using the NCBI Primer-BLAST tool and synthesized by Eurofins Genomics. The set of primer sequences used for real-time PCR are indicated in Supplementary Table S17, and the efficiencies of all primers used were between 90% and 105%. Next, qRT-PCR was conducted using a 20 μL reaction volume of the Maxima SYBR Green qPCR Master Mix (2×); each reaction contained a defined amount (0.02 μg) of cDNA and 10 pmol of gene-specific primers. The amplification protocol consisted of 30 cycles of denaturation at 95 °C for 10 s, followed by annealing/extension at 60 °C for 1 min. The relative gene expression levels were calculated using the comparative Ct method, and the relative RNA abundances were evaluated. To calculate the relative expression, we employed the 2^(−ΔΔCt)^ formula (VanGuilder et al., 2008) adjusted relative to controls (which was set at a value of 1). The expression of the target genes was calculated and normalized to that of glyceraldehyde-3-phosphate dehydrogenase (*GAPDH*) as an internal control (Boesenberg-Smith et al., 2012). Lastly, melt-curve analysis was performed to confirm the amplification specificity.

### 2.6 Computational study

#### 2.6.1 Network-pharmacology-based analysis

##### 2.6.1.1 Screening of *C. olitorius*-related target genes

The target genes associated with the compounds identified in the *C. olitorius* extract were predicted through chemical similarity analysis, pharmacophore modeling, and known protein–compound interaction data by searching the TCMSP database (https://old.tcmsp-e.com/index.php) (Ru et al., 2014), BATMAN-TCM platform (http://bionet.ncpsb.org.cn/batman-tcm/) (Kong et al., 2024), and SwissTargetPrediction Database (http://www.swisstargetprediction.ch/). The identified target genes were then standardized to their canonical names using the UniProt database (https://www.uniprot.org/) (UniProt, 2021).

##### 2.6.1.2 Screening of CHF-related target genes

Genes associated with CHF were gathered from the NCBI (https://www.ncbi.nlm.nih.gov), GeneCards (https://www.genecards.org/) (Fishilevich et al., 2017), and DisGeNET (https://www.disgenet.org/) databases using the keywords “congestive heart failure” or “myocardial injury,” with the species limited to “*Homo sapiens*”. The duplicate targets were eliminated, and intersecting proteins associated with both the compounds and disease were identified using InteractiVenn (https://www.interactivenn.net/) (Heberle et al., 2015) as potential targets of these components in CHF.

##### 2.6.1.3 Protein–protein interaction (PPI) network construction

A PPI network was constructed using STRING version 12.0 (https://string-db.org/) (Szklarczyk et al., 2023) and the target gene query list; the results were transferred to Cytoscape version 3.10.1 (Shannon et al., 2003) for visualization, modeling, and analysis of the molecular and genetic interaction networks (confidence score = 0.400), and the top-ten genes were analyzed using the CytoHubba plug-in.

#### 2.6.2 Molecular docking

The crystal structures of seven potential target proteins were retrieved from Protein Data Bank (PDB): AKT1 (PDB no. 4EJN), ACTB (PDB no. 2BTF), STAT3 (PDB no. 6NJS), IL-1β (PDB no. 6Y8M), TGF-β (PDB no. 6B8Y), TNF-α (PDB no. 2AZ5), and NF-κB (PDB no. 1SVC) (Berman et al., 2000). The input files of the identified compounds, protein structures, and co-crystallized ligands were prepared using AutoDockTools (Morris et al., 2009), and Open Babel v2.4 was used to process all the ligands (Trott and Olson, 2010). AutoDock Vina (Jaghoori et al., 2016) was used to dock the compounds to the investigated proteins. Grid boxes were defined with volumes not exceeding 27,000 Å^3^ and the exhaustiveness parameter set to 32, which are recommended for docking within relatively small search spaces. The center coordinates and dimensions (x, y, z) of each docking grid box were defined around the binding site of each co-crystallized ligand as follows: AKT1, center (14.2, 28.7, 8.5), dimensions 20 × 20 × 20 Å; ACTB, center (−3.1, 19.4, 16.8), dimensions 22 × 22 × 22 Å; STAT3, center (−22.5, 1.9, 35.6), dimensions 22 × 22 × 22 Å; IL-1β, center (24.3, 15.0, 22.1), dimensions 20 × 20 × 20 Å; TGF-β, center (−10.7, 8.3, −4.6), dimensions 22 × 22 × 22 Å; TNF-α, center (−2.3, 11.0, 30.8), dimensions 20 × 20 × 20 Å; and NF-κB, center (7.4, −12.3, 24.9), dimensions 22 × 22 × 22 Å. The binding sites were selected based on the locations of the co-crystallized ligands in each of the PDB structures, which correspond to the experimentally validated active or allosteric sites of each of the proteins, as reported in the respective structural studies deposited in PDB. To validate the docking protocols, the co-crystallized ligands were re-docked into the active sites of their respective protein structures. The re-docking validation yielded root mean-squared deviation (RMSD) values between the re-docked and original crystal positions of the proteins as follows: AKT1, 1.43 Å; ACTB, 1.78 Å; STAT3, 1.62 Å; IL-1β, 1.55 Å; TGF-β, 1.81 Å; TNF-α, 1.69 Å; and NF-κB, 1.47 Å. All RMSD values were below the accepted threshold of 2.0 Å, confirming that the docking protocols successfully reproduced the crystallographic binding positions and were therefore suitable for prospective docking of the tested compounds. The interactions between the docked compounds and key target proteins were then examined and interpreted using Discovery Studio Visualizer (version 21.1.0.2).

### 2.7 Statistical analysis

GraphPad Prism 6 (version 6.01) was used to perform the statistical computations in this work. The mean and standard deviation (SD) values for each group are shown, with n = 7 animals in each group. One-way ANOVA was used to evaluate the data, and *p* ≤ 0.05 based on Bonferroni’s multiple comparisons test was considered significant.

## 3 Results and discussion

### 3.1 Structure elucidation of the isolated compounds

Using various chromatographic techniques, eight compounds were isolated and purified as noted previously. The isolated compounds were classified according to their chromatographic behaviors and spectral data.

**FIGURE 1:**
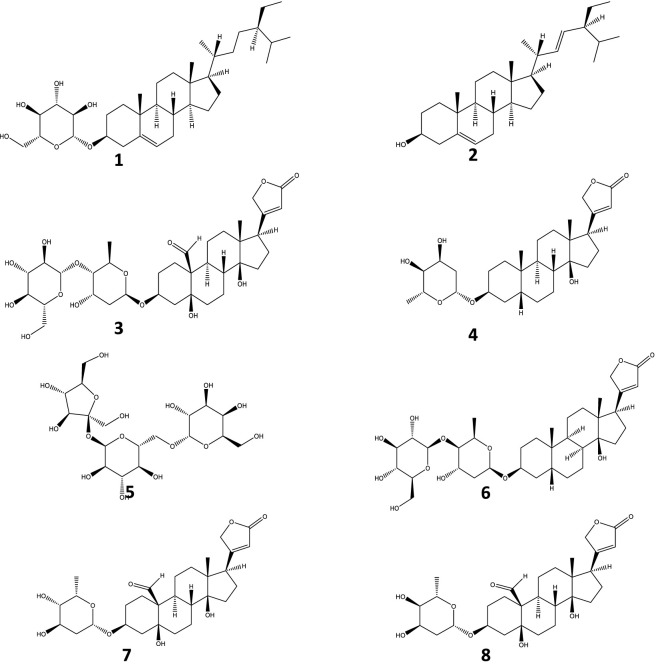
Structures of the compounds isolated from the seeds of *Corchorus olitorius*: (1) β-sitosterol-3-*O*-β-D-glucoside, (2) stigmasterol, (3) erysimoside, (4) evatromonoside, (5) raffinose, (6) coroloside, (7) corchoroside A, and (8) helveticoside.

The structures of the compounds were elucidated and identified by interpreting the spectral data with mass spectrometry (MS), ^1^H nuclear magnetic resonance (NMR), and ^13^C NMR, comparisons with literature, and following previously published data, all of which indicated that the identified compounds were previously reported in *Corchorus* species. The DCM and methanol extracts of *C. olitorius* seeds were extensively investigated to isolate five cardiac glycoside compounds, two steroidal compounds, and one trisaccharide sugar. Compounds 1–4 (Figure 1) were isolated from the DCM extract, while compounds 5–8 were obtained from the methanol extract. Compound 1 was identified as β-sitosterol-3-*O*-β-D-glucoside, which had previously been isolated from *C. olitorius* leaves and the vegetative parts of *Corchorus capsularis* (Hassan et al., 2019; Islam et al., 2013), and was discovered herein for the first time in *C. olitorius* seeds*.* Stigmasterol (compound 2) had been formerly identified from *C. olitorius* stems (Ragasa et al., 2016) but was isolated herein for the first time from the seeds. Erysimoside [strophanthidin 3-*O*-β-glucopyranosyl-(1→4)-β-digitoxopyranoside] (compound 3) (Hassan et al., 2019), evatromonoside [digitoxigenin 3-*O*-ß-D-digitoxopyranoside] (compound 4) (Nakamura et al., 1998), raffinose [galactose–glucose–fructose] (compound 5) (Hassan et al., 2019), coroloside [digitoxigenin 3-*O*-β-glucopyranosyl-(1→4)-β-boivinopyranoside] (compound 6) (Hassan et al., 2019), corchoroside A [strophanthidin 3-*O*-ß-D-boivinopyranoside] (compound 7) (Nakamura et al., 1998), and helveticoside [strophanthidin 3-*O*-ß-D-digitoxopyranoside] (compound 8) (Nakamura et al., 1998) have all been identified earlier from *C. olitorius* seeds (Nakamura et al., 1998).

### 3.2 Effects of crude extract of *C*. *olitorius* seeds on relative expression of *IL-1β*, *IL-6*, *TNF-α*, *NF-κB*, *ICAM-1*, and *MMP-9* in the cardiac tissues of DOX-treated rats

**FIGURE 2:**
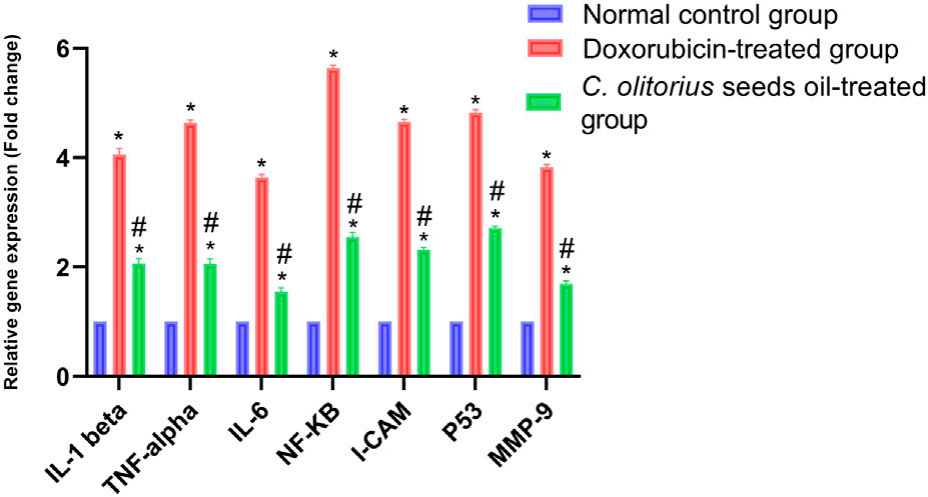
Relative gene expression in the cardiac tissues of different rat groups using qRT-PCR. After normalization to *GAPDH*, the data reflect the fold changes in expression compared to the control group. The data bars indicate mean ± SD. To ascertain the probability of substantial variations between the study groups, we performed a one-way ANOVA test followed by Bonferroni’s multiple comparisons test, with “*” being *p* ≤ 0.05 compared to the normal control group and “#” indicating *p* ≤ 0.05 compared to the doxorubicin-treated group.

We studied the relative expression of proinflammatory genes (*TNF-α*, *IL-1β*, and *IL-6*), the intercellular adhesion molecule (*ICAM-1*), matrix metalloproteinase 9 (*MMP-9*), tumor protein 53 (*P53*), and *NF-κB* in the cardiac tissues of the DOX-treated rats. The DOX-treated groups exhibited significant upregulation of all markers (*p* < 0.05) compared to the control group. The rats treated with the *C. olitorius* seed extract (*Group III*) displayed significant downregulation (*p* < 0.05) of the relative gene expression of the seven markers compared to the DOX-treated group (Figure 2).

The present study aims to ascertain the cardioprotective effects of *C. olitorius* seeds, and these changes are mediated by the modification of *NF-kB* implicated in inflammation and proliferation. *C. olitorius* seeds inhibited *NF-kB* activation triggered by DOX and also inhibited the *NF-kB*-dependent gene products associated with invasion (*MMP-9* and *ICAM-1*). Our findings show downregulation of *TNF-α*, *IL-1β*, and *IL-6* by the *C. olitorius* seeds, where all of these genes are known to be controlled by *NF-kB*. We also discovered that *C. olitorius* seeds decreased the expression of *MMP-9* and *ICAM-1* (Takada et al., 2005); this downregulation is correlated with the inhibition of proinflammatory markers (*TNF-α*, *IL-1β*, and *IL-6*) by *C. olitorius* seeds. *MMP-9* plays an important function in angiogenesis by regulating the breakdown of the extracellular matrix (Takada et al., 2005), whereas *p53* plays crucial roles in cell-cycle control (Waldman et al., 1997), DNA repair (Tokino et al., 1994), and induction of apoptosis (Polyak et al., 1997) in actively dividing cells. Several cellular stressors appear to impede *p53* breakdown, thereby increasing the protein level (Haupt et al., 1997; Kubbutat et al., 1997); activated *p53* protein works as a nuclear transcription factor to regulate different cell-cycle checkpoint genes, DNA repair, and apoptosis (Vogelstein et al., 2000). The functions of *p53* in the destruction of cardiac tissues are debatable. In culture models, *p53* plays a key role in hypoxia-mediated cell death (Long et al., 1997), although it does not seem necessary for hypoxia-induced apoptosis in a transgenic rat model (Bialik et al., 1997); *p53* was also discovered to be implicated in heart injury caused by stretch (Leri et al., 1998), pacing-induced heart failure, and activation of renin-angiotensin (Pierzchalski et al., 1997). *p53* is also known to control ROS generation (Johnson et al., 1996; Li et al., 1999). Therefore, the anti-apoptotic impacts of *p53* inhibition could be partially explained by the reduction of ROS generation by DOX. In conclusion, *NF-κB* and *p53* are central regulators of inflammation and apoptosis in DOX-induced cardiotoxicity. NF-κB activation promotes transcription of proinflammatory cytokines and adhesion molecules, while *p53* mediates apoptotic signaling in the cardiomyocytes. In our study, *C. olitorius* seed extract significantly downregulated the expression of both *NF-κB* and *p53*, suggesting attenuation of the inflammatory and apoptotic pathways. These findings, together with improvements in the ECG parameters, support the potential cardioprotective roles of *C. olitorius* seed extract. By integrating these results with computational analyses highlighting AKT1 and STAT3 as additional hub targets, we propose that the extract exerts its effects through coordinated modulation of the inflammatory and survival signaling pathways.

### 3.3 Effects of crude extract of *C. olitorius* seeds on ECG

**FIGURE 3:**
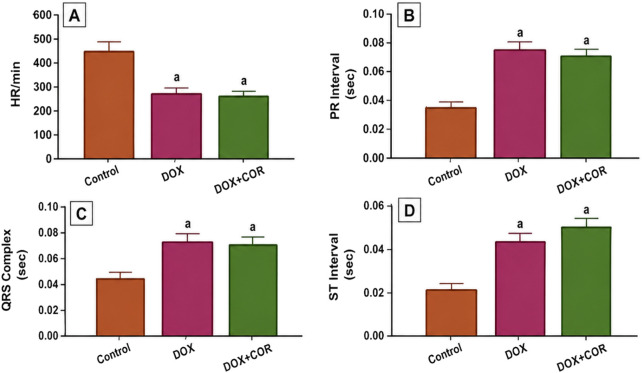
Effects of doxorubicin (DOX) and DOX plus (COR) on cardiac electrophysiological parameters compared to control. **(A)** Heart rate decreases in DOX-treated groups. **(B)** PR interval, **(C)** QRS complex duration, and **(D)** ST interval are increased in DOX-treated groups. Statistically significant differences versus control are indicated by “a” above the relevant bars.

The rats developed significant cardiotoxicity after DOX treatment, as shown by abnormal changes in their ECG, such as reduction in heart rate (HR), prolongation of the PR interval, prolonged QRS complex, and prolonged ST segment in the DOX group but not in the control animals. The cardiac-glycoside-like actions of *Corchorus* were evidenced by the slowed HR and prolonged ECG duration (Figure 3). These findings agree with previous reports that both DOX and cardiac glycosides slow the HR and prolong ECG duration (Morris et al., 2009; Trott and Olson, 2010).

### 3.4 Computational study

#### 3.4.1 Network-pharmacology-based analysis

Based on the cardiotonic activities of the cardiac glycosides in *Corchorus*, it is imperative to know the pathways and mechanisms through which these compounds act in treatment of CHF. First, we utilized network pharmacology analysis to map the interactions within biological networks; this reveals how drugs can affect various pathways and lead to therapeutic effects.

##### 3.4.1.1 Screening of *C. olitorius* extract-related target genes

A comprehensive set of 313 targets related to the compounds present in *C. olitorius* seed extract was identified using the TCMSP (Ru et al., 2014), BATMAN (Kong et al., 2024), and SwissTargetPrediction (Daina et al., 2019) databases. These identified genes were subsequently standardized to their official names through the UniProt database.

##### 3.4.1.2 Screening of CHF-related genes

**FIGURE 4:**
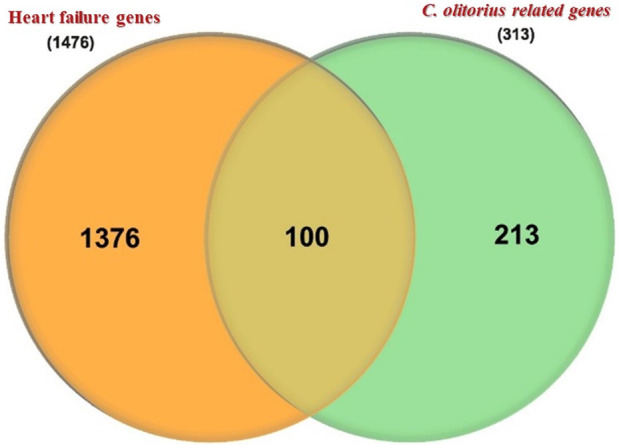
Venn diagram illustrates the overlap between heart failure-associated genes and *Corchorus olitorius*-related genes. A total of 1,476 genes were associated with heart failure, and 313 genes were related to C. olitorius, with 100 genes shared between the two datasets.

A total of 1,476 pharmacological targets associated with CHF were identified through a comprehensive search across the NCBI, GeneCards, and DisGeNET databases by employing specific search terms (“congestive heart failure” or “myocardial injury”) and restricting the results to “*Homo sapiens*.” An interactive Venn diagram was produced to compare the targets modulated by compounds obtained from *C. olitorius* extract with potential CHF-related targets from each database. This revealed a total of 100 genes that were common with CHF targets from the three databases after removing duplicates (Figure 4).

##### 3.4.1.3 PPI network construction

**FIGURE 5:**
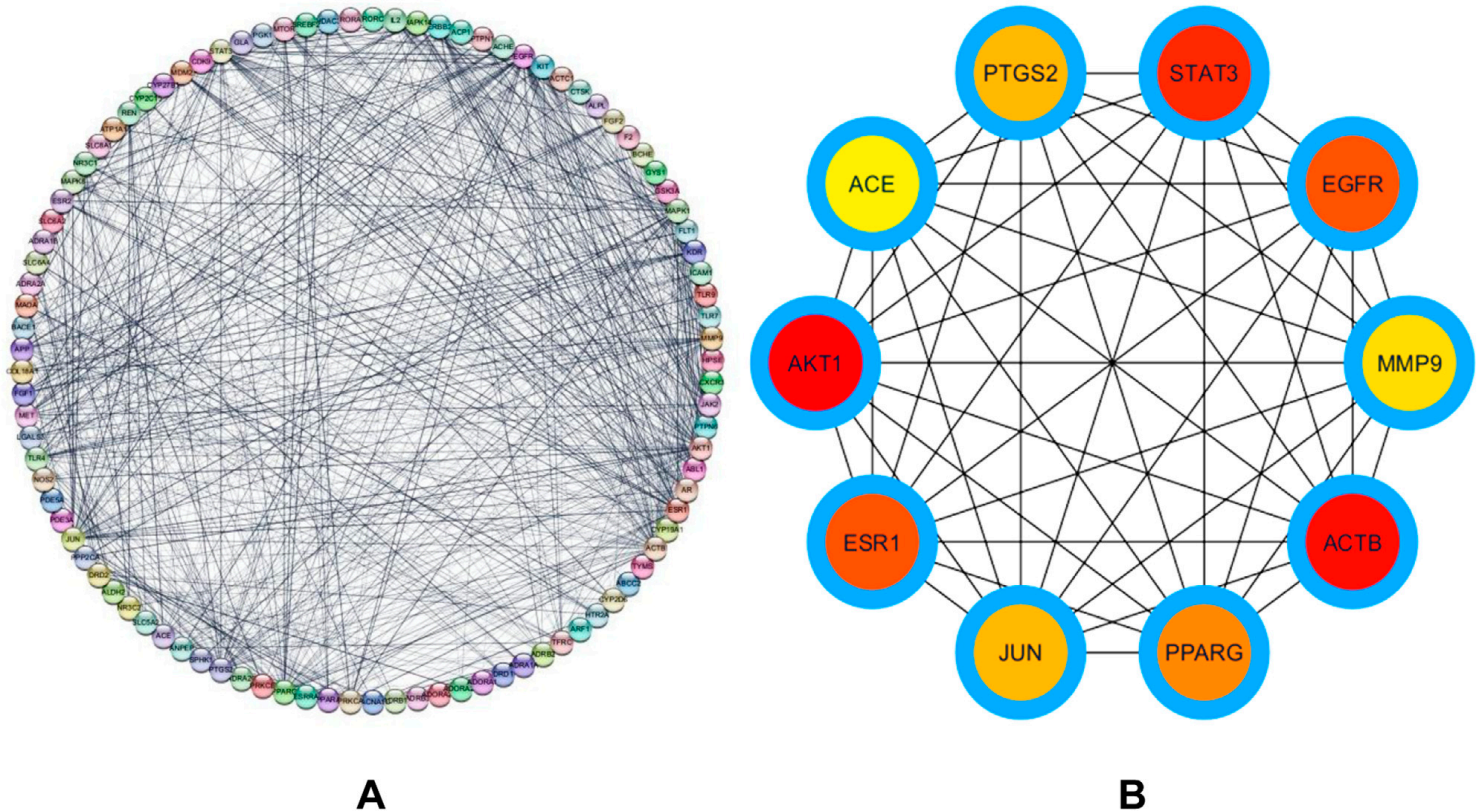
Protein–protein interaction (PPI) network analysis of shared genes. **(A)** Full PPI network showing all identified proteins as nodes connected by interaction edges. **(B)** Simplified subnetwork highlighting the top ten hub proteins, color-coded in red, orange, and yellow and outlined with a blue ring, with edges indicating their interactions.

The 100 shared targets recognized using the Venn interaction analysis were introduced to the STRING database to investigate their interrelationships. Then, a PPI network was established using Cytoscape v3.10.2, which resulted in 97 nodes (following removal of three unconnected nodes), 903 edges, and an average node connectivity of 18.61, as shown in Figure 5A. Consequently, the CytoHubba plug-in was used to pinpoint the top-ten most prominent genes, as determined by their connectivity degree within the network (Figure 5B). These genes are *AKT1*, *ACTB*, *STAT3*, *EGFR*, *ESR1*, *PPARG*, *PTGS2*, *JUN*, *MMP-9*, and *ACE*. The topological parameters, such as node degree, betweenness, and closeness, are summarized for each protein in Table 1.

**TABLE 1:** Topological parameters of the top ten hub genes.

No.	Name	Target	Degree	Betweenness	Closeness
1	RAC-α serine/threonine-protein kinase	*AKT1*	67	0.1379	0.7619
2	Actin	*ACTB*	58	0.0860	0.7111
3	Signal transducer and activator of transcription 3	*STAT3*	52	0.0456	0.6761
4	Epidermal growth factor	*EGFR*	49	0.0361	0.6667
5	Estrogen receptor	*ESR1*	49	0.0513	0.6667
6	Peroxisome proliferator-activated receptor gamma	*PPARG*	48	0.0457	0.6575
7	Prostaglandin G/H synthase 2	*PTGS2*	45	0.0337	0.6486
8	Transcription factor Jun	*JUN*	45	0.0226	0.6400
9	Matrix metalloproteinase 9	*MMP-9*	43	0.0295	0.6400
10	Angiotensin-converting enzyme	*ACE*	40	0.0574	0.6275

#### 3.4.2 Molecular docking

Molecular docking was conducted to study the two isolated compounds from *C. olitorius* seed extract that exhibited potential cardiotonic properties by interacting with the target proteins. First, we selected the top-three hub genes (*AKT1*, *ACTB*, and *STAT3*) identified from the network pharmacology analysis along with key proinflammatory and inflammatory cytokines (IL-1β, TGF-β, TNF-α, and NF-κB) for the docking study. To successfully confirm the docking methodology, we re-docked the co-crystallized ligands while ensuring that the RMSD between the crystal and docked positions was less than 2 Å. The isolated compounds were then docked to these genes; Table 2 displays the docking scores for the seven targets. We then compared the docking scores of the co-crystallized ligands and tested compounds, with lower scores corresponding to stronger binding affinities.

**TABLE 2:** Docking scores of the tested compounds.

Compound	Docking score (kcal/mol)						
	AKT1 (4EJN)	ACTB (2BTF)	STAT3 (6NJS)	IL-1β (6Y8M)	TGF-β (6B8Y)	TNF-α (2AZ5)	NF-κB (1SVC)
1	-7.728	-3.397	-3.553	-2.866	-5.543	-5.181	-3.464
2	-6.687	-4.301	-2.955	*ND	-6.865	-3.297	-2.755
3	ND	-4.271	-4.521	-3.398	ND	-3.783	-4.524
4	ND	-3.156	-4.434	-4.801	-5.349	-4.010	-3.776
5	-7.634	-5.140	-4.345	-3.950	-6.587	-3.978	-4.633
6	-7.761	-4.597	-6.349	-5.012	-6.606	-4.896	-6.382
7	-6.951	-4.282	-5.502	-4.110	-6.467	-4.574	-5.076
8	-7.236	-2.445	-2.782	ND	-6.556	-3.259	-3.873
Co-crystallized ligand	-12.06	—	-10.01	-5.62	-11.69	-5.173	—

*ND, not detected.

According to the docking results, compound 6 (coroloside) showed notable docking scores for several gene targets, although its relative performance varied depending on the protein. For AKT1, compound 6 achieved a docking score of −7.761 kcal/mol. Notably, the docking scores of compound 1 (−7.699 kcal/mol) and compound 5 (−7.712 kcal/mol) were very close to that of compound 6, indicating comparable binding affinities for these targets. For ACTB, compound 6 displayed a significant binding affinity with a score of −4.597 kcal/mol, which was slightly lower than that of compound 5 (−5.140 kcal/mol), demonstrating that compound 5 had a stronger affinity toward ACTB than compound 6. The binding results for STAT3 further underscored the efficacy of compound 6, which had the lowest docking score of −6.349 kcal/mol, indicating the strongest interaction among the tested compounds.

In addition to these targets, compound 6 demonstrated a superior binding affinity with IL-1β, achieving a docking score of −5.012 kcal/mol that was the highest among all compounds tested for this target. For TGF-β, compound 6 showed a competitive docking score of −6.606 kcal/mol, second only to compound 2. The docking score for compound 6 with TNF-α was −4.896 kcal/mol, emphasizing its strong binding potential. Finally, compound 6 showed the best performance for NF-κB with a docking score of −6.382 kcal/mol, surpassing all other compounds.

**FIGURE 6:**
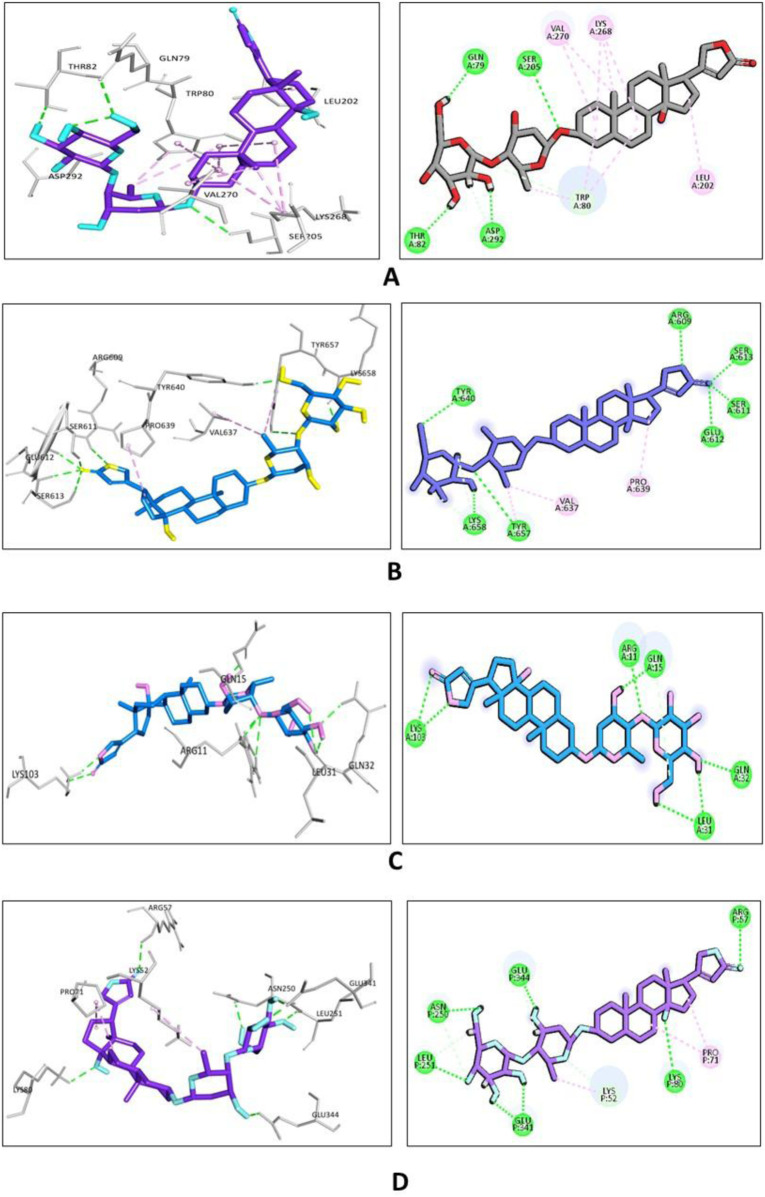
Molecular docking analysis of compound 6 with the top-ranked target proteins: **(A)** AKT1, **(B)** STAT3, **(C)** IL-1B, and **(D)** NF-κB. For each target, the left panel shows the three-dimensional binding pose with key interacting amino acid residues and hydrogen bonds, while the right panel shows the corresponding two-dimensional interaction diagram highlighting key residues and interaction sites with colored bonds and labeled atoms.

In conclusion, compound 6 (coroloside) demonstrated favorable docking scores for most of the investigated targets, particularly exhibiting the lowest binding energies toward STAT3, IL-1β, TNF-α, and NF-κB. However, it is important to note that compound 5 showed stronger affinity toward ACTB and compound 2 outperformed compound 6 with TGF-β, while the differences among compounds 1, 5, and 6 at AKT1 were marginal. Overall, these results suggest that coroloside displays broad-spectrum binding potential, most notably with AKT1, STAT3, IL-1β, and NF-κB, supporting its candidacy as a multitarget agent; however, other compounds also warrant attention for specific protein targets (Figure 6).

#### 3.4.3 MD studies

MD simulations were performed to assess the stability of compound 6 that exhibited the highest docking score in a complex with AKT1 and to provide insights into the behavior and stability of the complex.

**FIGURE 7:**
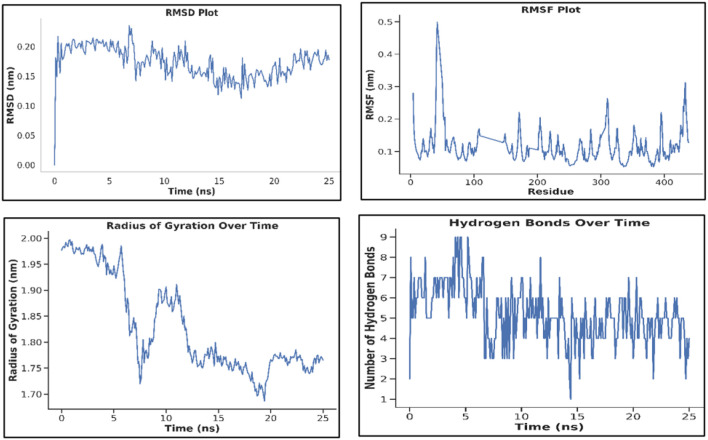
Molecular dynamics simulation analysis of compound 6 in the active site of AKT1 over 25 ns. (Top left) Root-mean-square deviation (RMSD) versus time, showing an initial rise followed by stabilization around 0.17–0.20 nm. (Top right) Root-mean-square fluctuation (RMSF) per residue, with a prominent peak near residue 50. (Bottom left) Radius of gyration (Rg) over time, showing a stepwise decline from 2.00 to approximately 1.75 nm. (Bottom right) Number of hydrogen bonds over time, fluctuating between 1 and 9 with a decreasing trend after several peaks.

The RMSD plot in Figure 7 indicates that the complex experiences structural changes within the initial few nanoseconds, achieving stability with an RMSD value of approximately 0.2 nm by approximately 5 ns. This implies that the system reaches equilibrium relatively quickly and retains its structural consistency throughout the remaining duration of the 25-ns simulation time. The stable RMSD value confirms that the interaction between compound 6 and AKT1 remains steady over the duration of the simulation.

Furthermore, examination of the root mean-squared fluctuation (RMSF) plot reveals the flexibility of specific residues within AKT1. The fluctuation profile shows that most regions of the protein exhibit low levels of flexibility, with only a few peaks being observed near residue numbers 50 and 400, which could correspond to the flexible loop regions. However, the lower fluctuations in other areas suggest that most parts of AKT1 remain structurally stable in the presence of compound 6. This implies that compound 6 does not provoke major conformational shifts in the essential structural components of the protein (Figure 7).

The radius of gyration (Rg) plot provides additional evidence of the stability of the system, showing a gradual drop from approximately 1.95 nm to 1.75 nm over the simulation duration. This reduction in Rg indicates that the protein–ligand complex becomes more compact over time, suggesting that the interaction between compound 6 and AKT1 promotes a more tightly packed and stable conformation. A more compact complex often reflects a stronger interaction between the ligand and target protein, which could have positive implications for the binding affinity and efficacy of compound 6 as a potential inhibitor.

Finally, analysis of the hydrogen bonds over time demonstrates consistent hydrogen bonding interactions between compound 6 and AKT1 throughout the simulation. The number of hydrogen bonds fluctuates between four and eight, evidencing that compound 6 maintains stable and significant interactions with the protein. Hydrogen bonding is crucial in stabilizing the protein–ligand complex, and the persistence of these interactions further supports the notion that compound 6 is a promising candidate for targeting AKT1.

Overall, the MD simulations indicate that compound 6 produces a stable, compact, and well-interacting complex with AKT1, suggesting its potential as a potent AKT1 inhibitor for further development.

## 4 Conclusion

This study provides compelling evidence that *C. olitorius* seeds hold significant potential as a prophylactic agent against DOX-induced cardiotoxicity, given the presence of isolated cardiac glycosides and other compounds in the crude extract from the seeds. Eight compounds were successfully isolated and identified from the extract, including the cardiac glycosides erysimoside (compound 3), evatromonoside (compound 4), coroloside (compound 6), corchoroside A (compound 7), and helveticoside (compound 8), along with β-sitosterol-3-*O*-β-D-glucoside (compound 1), stigmasterol (compound 2), and raffinose (compound 5). The crude extract of *C. olitorius* seeds demonstrated remarkable cardioprotective effects by significantly downregulating proinflammatory genes (*TNF-α*, *IL-1β*, *IL-6*), adhesion molecules (*ICAM-1*), *MMP-9*, and *p53*, all of which are implicated in DOX-induced cardiac damage. Furthermore, the extract effectively normalized adverse ECG changes induced by DOX, indicating a positive impact on cardiac function. We used network pharmacology analysis to identify 100 common targets between the compounds isolated from *C. olitorius* extract and markers implicated in CHF, with the key hub genes being *AKT1*, *ACTB*, *STAT3*, *EGFR*, *ESR1*, *PPARG*, *PTGS2*, *JUN*, *MMP*-*9*, and *ACE*. Molecular docking studies suggested that coroloside (compound 6) has a favorable broad binding profile toward several relevant targets, particularly STAT3, IL-1β, and NF-κB. However, the docking analysis also indicated that other isolated compounds may contribute to the observed activity since compound 5 showed the best predicted binding toward ACTB, compound 2 toward TGF-β, and compounds 1, 5, and 6 exhibited close comparable docking scores toward AKT1. The stability and strong interaction of compound 6 with AKT1 were further corroborated by MD simulations. These findings collectively underscore the therapeutic promise of *C. olitorius* seeds and their constituents in mitigating DOX-induced cardiac injury. In the future, studies will focus on evaluating the individual cardioprotective effects of the isolated compounds, particularly coroloside, in preclinical models of DOX-induced cardiotoxicity to confirm their efficacy and understand the specific action mechanisms. Advanced research is needed to uncover the precise molecular and cellular pathways by which *C. olitorius* compounds modulate inflammation, oxidative stress, and apoptosis in the context of DOX-induced cardiotoxicity; this could involve omics technologies (e.g., proteomics and metabolomics) to identify additional biomarkers and targets. Investigating the structure–activity relationships of the isolated cardiac glycosides could lead to the design and synthesis of novel, more potent, and selective derivatives with improved pharmacological properties and reduced potential side effects.

Future works will also include more comprehensive validation of the extract and/or its constituents in terms of cardiac injury and protection. These additional assays were beyond the scope of the current study but are planned for future work to build a more complete picture of the comprehensive effects of the extract.

## Data Availability

The original contributions presented in the study are included in the article/Supplementary Material; further inquiries can be directed to the corresponding authors.
