# Supplementary material for: Integrated in vivo and in silico validation of Corchorus olitorius seed-mediated cardioprotection against doxorubicin-induced cardiotoxicity via network pharmacology, molecular docking, and molecular dynamics
Source: Front Chem. 2026 Jul 30;14:1810825. doi: 10.3389/fchem.2026.1810825 (PMC13469431; doi:10.3389/fchem.2026.1810825)
Supplement: Supplementary file 1 [file DataSheet1.docx]

**Integrated *In Vivo* and *In Silico* Validation of *Corchorus olitorius* Seed-Mediated Cardioprotection against Doxorubicin-Induced Cardiotoxicity via Network Pharmacology, Molecular Docking, and Dynamics**

Fatma Mohamed Abd El-Mordy ^a^, Sherif A. Maher ^b^, Omar Y Tammam ^b^, Mahmoud A. Elrehany ^c, d^, Mohamed Hisham ^e^, Remon R. Rofaeil ^f^, Mostafa E. Rateb ^g^, Hesham F. Oraby ^h^, Mahmoud A. A. Ibrahim ^i,j^, Ahmed M. Shawky ^k,^ Gerhard Bringmann ^l^, Usama Ramadan Abdelmohsen ^m,n*^, Nourhan Hisham Shady ^n,o*^

^a^ Department of Pharmacognosy and Medicinal Plants, Faculty of Pharmacy (Girls), Al-Azhar University, Cairo, 11754, Egypt; [fatmamohammed.pharmg@azhar.edu.eg](mailto:fatmamohammed.pharmg@azhar.edu.eg); ORCID: <https://orcid.org/0000-0002-5554-2064>

^b^ Department of Biochemistry, Faculty of Pharmacy, New Valley University, El-kharga, Egypt; [sherif.ali87@Pha.nvu.edu.eg](mailto:sherif.ali87@Pha.nvu.edu.eg), Omar.Tammam93@Pha.nvu.edu.eg

^c^ Department of Biochemistry, Faculty of Medicine, Minia University, Minia 61519, Egypt; elrehany1963@mu.edu.eg

^d^ Department of Biochemistry, Faculty of Pharmacy, Deraya University, Universities Zone, New Minia City 61111, Egypt

^e^ Department of Pharmaceutical Chemistry, Faculty of Pharmacy, Deraya University, New-Minia 61111, Egypt; [dr.mohamedhisham@deraya.edu.eg](mailto:dr.mohamedhisham@deraya.edu.eg).

^f^ Department of Pharmacology, Deraya University, Universities Zone, New Minia City 61111, Egypt; remon.roshdy@deraya.edu.eg

^g^ Natural and Medical Sciences Research Center, University of Nizwa, P. O. Box 33, Birkat Al Mauz, Nizwa, Oman; [ratebnplab@gmail.com](mailto:ratebnplab@gmail.com)

^h^ Biotechnology/ Molecular Biology and Biomedical Sciences Programs Faculty of Basic and Applied Science Egypt-Japan University of Science and Technology (EJUST) New Borg El-Arab City, 21934 Alexandria, Egypt; [hesham.oraby@ejust.edu.eg](mailto:hesham.oraby@ejust.edu.eg)

^i^ School of Health Sciences, University of KwaZulu-Natal, Westville Campus, Durban 4000, South Africa m.ibrahim@compchem.net

^j^ Department of Supportive Requirements, University of Technology and Applied Sciences, Nizwa 611, Sultanate of Oman

^k^ Department of Chemistry, Faculty of Science, Umm Al-Qura University, 21955 Makkah, Saudi Arabia

amesmail@uqu.edu.sa

^l^ Institute of Organic Chemistry, University of Würzburg, Am Hubland, 97074 Würzburg, Germany; [gerhard.bringmann@uni-wuerzburg.de](mailto:gerhard.bringmann@uni-wuerzburg.de); ORCID: <https://orcid.org/0000-0002-3583-593>.

^m^ Department of Pharmacognosy, Faculty of Pharmacy, Minia University, Minia 61519, Egypt; usama.ramadan@mu.edu.eg

^n^ Department of Pharmacognosy, Faculty of Pharmacy, Deraya University, Universities Zone, New Minia City 61111, Egypt. [Norhan.shady@deraya.edu.eg](mailto:Norhan.shady@deraya.edu.eg)

^o^ Center for Entrepreneurship and Sustainability Research, Deraya University, Universities Zone, New Minia City, 61111, Egypt

*** Correspondence:** [usama.ramadan@mu.edu.eg,](mailto:usama.ramadan@mu.edu.eg,Nourhan) [Norhan.shady@deraya.edu.eg](mailto:Norhan.shady@deraya.edu.eg)

**Table of Contents**

| **Table S1:** | ^13^C-NMR chemical shifts of compounds **3**_,_ **4**_,_ **6**_,_ **7**_,_ and **8**_._ |
| --- | --- |
| **Table S2:** | ^1^H-NMR and ^13^C-NMR chemical shifts of compound **5** |
| **Figure S1:** | ^1^H-NMR spectrum of compound **1** in CDCl_3_ (600 MHz) |
| **Figure S2:** | ^13^C-NMR spectrum of compound **1** in CDCl_3_ (150 MHz) |
| **Figure S3:** | ^1^H-NMR spectrum of compound **2** in CDCl_3_ (600 MHz) |
| **Figure S4:** | ^13^C-NMR spectrum of compound **2** in CDCl_3_ (150 MHz) |
| **Figure S5:** | ^1^H-NMR spectrum of compound **3** in DMSO-*d*_6_ (600 MHz) |
| **Figure S6:** | ^13^C-NMR spectrum of compound **3** in DMSO (150 MHz) |
| **Figure S7:** | ^1^H-NMR spectrum of compound **4** in DMSO (600 MHz) |
| **Figure S8:** | ^13^C-NMR spectrum of compound **4** in DMSO (150 MHz) |
| **Figure S9:** | ^1^H-NMR spectrum of compound **5** in DMSO (600 MHz) |
| **Figure S10:** | ^13^C-NMR spectrum of compound **5** in DMSO (150 MHz) |
| **Figure S11:** | ^1^H-NMR spectrum of compound **6** in DMSO (600 MHz) |
| **Figure S12:** | ^13^C-NMR spectrum of compound **6** in DMSO (150 MHz) |
| **Figure S13:** | ^1^H-NMR spectrum of compound **7** in DMSO (600 MHz) |
| **Figure S14:** | ^13^C-NMR spectrum of compound **7** in DMSO (150 MHz) |
| **Figure S15:** | ^1^H-NMR spectrum of compound **8** in DMSO (600 MHz) |
| **Figure S16:** | ^13^C-NMR spectrum of compound **8** in DMSO (150 MHz) |
| **Table S 3:** | Primers used for real-time PCR |

**Methods**

***β-Sitosterol-3-O-β-D-glucoside* (1):** White powder; m/z 576.8; ^1^H-NMR (600 MHz, CDCl_3_): δH 5.34 (m, 1H), 4.21 (H-1ˋ), 3.60 (m, 1H), 0.83-1.30 (5×3H); ^13^C-NMR (150 MHz, CDCl_3_): δc 140.36 (C-5), 138.46 (C-22), 129.24, 121.87, 101.29 (C-1ˋ), 76.79 (C-3ˋ), 76.31(C-5ˋ), 73.46 (C-2ˋ), 71.97, 70.05 (C-4ˋ), 61.13 (C-6ˋ), 56.92, 56.83, 50.22, 45.88, 42.42, 42.41, 38.98, 37.34, 36.80, 34.10, 31.82 (2), 32.05, 29.17, 28.40, 27.33, 25.06, 23.22, 21.16, 19.94, 19.47, 19.11, 12.08, 11.96 ppm (Supplementary Materials, Figures S1, S2).

***Stigmasterol* (2):** White needles; m/z 412; ^1^H-NMR (600 MHz, CDCl_3_): δH 5.33 (m,1H), 5.01 (dd, *J* = 8.8, 15.2 Hz, 1H) and 5.13 (dd, *J* = 8.8, 15.2 Hz, 1H) 3.54 (m, 1H), 0.65 -1.27 (m, 5× 3H); ^13^C-NMR (150 MHz, CDCl_3_): δc 140.36, 138., 130.13, 122.27, 71.97, 56.81, 56.12, 51.29, 50.20, 42.39 (2), 40.46, 38.95, 37.33, 36.79, 32.04 (2), 31.91 (2), 29.14, 25.72, 24.52, 22.81, 21.14 (2), 19.94, 18.87, 12.07, 11.95 ppm (Supplementary Materials, Figures S3, S4).

***Erysimoside* (3):** White amorphous powders. MS m/z: 696 [M±H]. ^13^C-NMR: see Table S1 (Supplementary Materials, Figures S5, S6).

***Evatromonoside* (4):** White amorphous powder. MS m/z: 503 [M±H]. ^1^H-NMR (600 MHz, DMSO): 0.85 (3H, s, H3-18), 1.08 (1H,s, H3-19), 1.23 (3H, d, J = 6.0 Hz, dig H3-6), 2.96 (1H, m, H-17), 4.05 (1H, m, H-3), 4.90, 5.02 (each 1H, dd, J = 2.0, 18.0 Hz, H2-21), 4.93 (1H, dd, J = 2.0, 9.5 Hz, dig H-1), 5.88 (1H, s, H-22). ^13^C-NMR: see Table S1 (Supplementary Materials, Figures S7, S8).

***Raffinose* (5):** Table S2 shows ^1^H-nuclear magnetic resonance (600 MHz, DMSO), and ^13^C-nuclear magnetic resonance (150 MHz, DMSO) chemical shifts of **raffinose** (**5**) (Supplementary Materials, Figures S9, S10).

***Coroloside* (6):** White amorphous powder. m/z: 665 [M±H]. ^1^H-NMR (600 MHz, DMSO): δH 0.82 (3H, s, H3-18), 1.08 (3H, s, H3-19), 1.20 (3H, d, J = 6.5 Hz, boi H3-6), 2.98 (1H, m, H-17), 3.64 (1H, dd, J = 5.5, 12.0 Hz, glc H-6a), 3.87 (1H, dd, J = 2.0, 12.0 Hz, glc H-6b), 4.03 (1H, m, H-3), 4.36 (1H, d, J = 7.5 Hz, glc H-1), 4.85 (1H, dd, J = 2.0, 10.0 Hz, boi H-1), 4.92, 5.04 (each 1H, dd, J = 2.0, 18.5 Hz, H2-21), 5.87 (1H, s, H-22). ^13^C-NMR: see Table S1 (Supplementary Materials, Figures S11, S12)

***Corchoroside* (7):** White amorphous powder. m/z: 533 [M±H]. ^1^H-NMR (600 MHz, DMSO): δH 0.86 (3H, s, H3-18), 1.25 (3H, d, J = 6.5 Hz, boi H3-6), 2.69 (1H, m, H-17), 3.18 (1H, m, boi H-4), 3.95 (1H, m, boi H-5), 3.96 (1H, m, boi H-3), 4.18 (1H, m, H-3), 4.88 (1H, dd, J = 2.0, 10.0 Hz, boi H-1), 4.90, 5.02 (each 1H, dd, J = 2.0, 18.5 Hz, H2-21), 5.88 (1H, s, H-22), 10.01 (1H, s, H3-19). ^13^C-NMR: see Table S1 (Supplementary Materials, Figures S13, S14).

***Helveticoside* (8):** White amorphous powder. m/z: 533 [M±H]. ^1^H-NMR (600 MHz, DMSO): δH 0.86 (3H, s, H3-18), 1.23 (3H, d, J = 6.5 Hz, dig H3-6),2.90 (1H, m, H-17), 3.14 (1H, dd, J = 3.0, 9.5 Hz, dig H-4), 3.89 (1H, dq, J = 6.5, 9.5 Hz, dig H-5), 4.07 (1H, m, dig H-3), 4.18 (1H, m, H-3), 4.90, 5.02 (each 1H, dd, J = 2.0, 18.5 Hz, H2-21), 4.94 (1H, dd, J = 2.0, 10.0 Hz, dig H-1), 5.88 (1H, s, H-22), 10.01 (1H, s, H3-19). ^13^C-NMR: see Table S1 (Supplementary Materials, Figures S15, S16).

The following Table **S1** shows ^13^C-nuclear magnetic resonance (150 MHz, DMSO) chemical shifts of compounds **3_,_ 4_,_ 6_,_ 7_,_** and **8_._**

**Table S1:** ^13^C-NMR chemical shifts of compounds **3_,_ 4_,_ 6_,_ 7**_,_ and **8_._**

| ***Compound*** | **3** | **4** | **6** | **7** | **8** |
| --- | --- | --- | --- | --- | --- |
| ***Aglycone*** | | | | | |
| ***1*** | ***25.08*** | ***31.40*** | ***31.40*** | ***25.43*** | ***25.42*** |
| ***2*** | ***26.76*** | ***27.55*** | ***27.50*** | ***25.9*** | ***25.9*** |
| ***3*** | ***75.61*** | ***74.49*** | ***73.80*** | ***76.82*** | ***76.88*** |
| ***4*** | ***36.43*** | ***31.02*** | ***31.03*** | ***37.35*** | ***37.39*** |
| ***5*** | ***74.25*** | ***38.01*** | ***38.05*** | ***75.30*** | ***75.30*** |
| ***6*** | ***36.43*** | ***28.02*** | ***26.93*** | ***37.1*** | ***37.1*** |
| ***7*** | ***22.44*** | ***22.96*** | ***22.60*** | ***23.2*** | ***23.2*** |
| ***8*** | ***41.47*** | ***41.47*** | ***41.57*** | ***42.5*** | ***42.6*** |
| ***9*** | ***39.08*** | ***36.9*** | ***36.9*** | ***40.4*** | ***40.4*** |
| ***10*** | ***55.09*** | ***35.22*** | ***36.3*** | ***56.22*** | ***56.22*** |
| ***11*** | ***19.0*** | ***22.4*** | ***21.62*** | ***18.9*** | ***18.9*** |
| ***12*** | ***40.59*** | ***40.64*** | ***40.50*** | ***40.5*** | ***40.5*** |
| ***13*** | ***50.35*** | ***49.14*** | ***49.75*** | ***49.92*** | ***49.92*** |
| ***14*** | ***84.04*** | ***84.5*** | ***84.34*** | ***84.48*** | ***84.48*** |
| ***15*** | ***31.76*** | ***32.4*** | ***33.50*** | ***34.39*** | ***34.39*** |
| ***16*** | ***26.75*** | ***28.0*** | ***28.10*** | ***26.9*** | ***26.93*** |
| ***17*** | ***49.59*** | ***49.98*** | ***49.92*** | ***50.56*** | ***50.56*** |
| ***18*** | ***16.01*** | ***16.4*** | ***16.16*** | ***16.16*** | ***16.16*** |
| ***19*** | ***209.29*** | ***24.3*** | ***24.3*** | ***208.91*** | ***208.92*** |
| ***20*** | ***176.28*** | ***176.5*** | ***177.35*** | ***176.1*** | ***176.07*** |
| ***21*** | ***75.2*** | ***75.4*** | ***75.3*** | ***75.27*** | ***75.26*** |
| ***22*** | ***116.86*** | ***116.96*** | ***116.75*** | ***116.75*** | ***116.74*** |
| ***23*** | ***174.45*** | ***174.3*** | ***174.42*** | ***174.5*** | ***174.5*** |
| **Sugar I** | | | | | |
| ***1*** ˋ | ***97.38*** | ***99.0*** | ***98.4*** | ***99.0*** | ***98.3*** |
| ***2*** ˋ | ***38.82*** | ***39.9*** | ***35.0*** | ***34.39*** | ***37.35*** |
| ***3*** ˋ | ***68.53*** | ***69.3*** | ***68.46*** | ***70.29*** | ***69.60*** |
| ***4*** ˋ | ***84.04*** | ***73.68*** | ***75.56*** | ***70.88*** | ***74.2*** |
| ***5*** ˋ | ***69.0*** | ***71.8*** | ***70.88*** | ***70.43*** | ***70.88*** |
| ***6*** ˋ | ***18.57*** | ***18.18*** | ***17.53*** | ***16.16*** | ***18.59*** |
| **Sugar II** | | | | | |
| ***1***" | ***104.83*** |  | ***102.40*** |  |  |
| ***2***" | ***75.61*** |  | ***74.9*** |  |  |
| ***3***" | ***77.59*** |  | ***77.57*** |  |  |
| ***4***" | ***70.81*** |  | ***70.88*** |  |  |
| ***5***" | ***77.28*** |  | ***78.0*** |  |  |
| ***6***" | ***61.90*** |  | ***61.93*** |  |  |

**Table S2:** ^1^H-NMR and ^13^C -NMR chemical shifts of compound **5**

|  | δ ^13^C | δ ^1^H |
| --- | --- | --- |
| Fructose |  |  |
| 1-CH_2_OH | 64.35 | 3.66 |
| 2 | 105.31 | - |
| 3 | 75.38 | 4.12 |
| 4 | 70.52 | 3.78-3.70 |
| 5 | 71.45 | 3.81-3.78 |
| 6-CH_2_OH | 62.75 | 3.70-3.67 |
| Glucose |  |  |
| 1 ˋ | 92.12 | 5.14 |
| 2 ˋ | 73.10 | 3.45 |
| 3 ˋ | 74.64 | 3.78 |
| 4 ˋ | 71.97 | 3.40 |
| 5 ˋ | 73.31 | 4.07 |
| 6 ˋ | 69.32 | 3.86 |
| Galactose |  |  |
| 1" | 99.61 | 4.89 |
| 2" | 82.88 | 3.78-3.70 |
| 3" | 79.25 | 4.12 |
| 4" | 71.07 | 3.90 |
| 5" | 72.46 | 3.85 |
| 6" | 63.11 | 3.82-3.78 |

**Figure S1:** ^1^H-NMR spectrum of compound **1** in CDCl_3_ (600 MHz)

**Figure S2:** ^13^C-NMR spectrum of compound **1** in CDCl_3_ (150 MHz)

**[M-H] ‾**

**Figure S3:** ^1^H-NMR spectrum of compound **2** in CDCl_3_ (600 MHz)

**Figure S4:** ^13^C-NMR spectrum of compound **2** in CDCl_3_ (150 MHz)

**Figure S5:** ^1^H-NMR spectrum of compound **3** in DMSO (600 MHz)

**Figure S6:** ^13^C-NMR spectrum of compound **3** in DMSO (150 MHz)

**Figure S7:** ^1^H-NMR spectrum of compound **4** in DMSO (600 MHz)

**Figure S8:** ^13^C-NMR spectrum of compound **4** in DMSO (150 MHz)

**Figure S9:** ^1^H-NMR spectrum of compound **5** in DMSO (600 MHz)

**Figure S10:** ^13^C-NMR spectrum of compound **5** in DMSO (150 MHz)

**Figure S11:** ^1^H-NMR spectrum of compound **6** in DMSO_3_ (600 MHz)

**Figure S12:** ^13^C-NMR spectrum of compound **6** in DMSO (150 MHz)

**Figure S13:** ^1^H-NMR spectrum of compound **7** in DMSO (600 MHz)

**Figure S14:** ^13^C-NMR spectrum of compound **7** in DMSO (150 MHz)

**Figure S15:** ^1^H-NMR spectrum of compound **8** in DMSO (600 MHz)

**Figure S16:** ^13^C-NMR spectrum of compound **_8_** in DMSO (150 MHz)

**Materials and Methods**

**Table S 3.** Primers used for real-time PCR.

| **Name of gene** | **Sequence 5' to 3'** |
| --- | --- |
| ***TNF-α*** | **Forward** **CCT CTC TGC CAT CAA GAG CC**  **Reverse** **GGC TGG GTA GAG AAC GGA TG** |
| ***IL-1β*** | **Forward** **GTG ATG AAA GAC GGC ACA CC**  **Reverse** **TCC TGG GGA AGG CAT TAG GA** |
| ***NF-KB*** | **Forward** **CAG CAG ATG GCC CAT ACC TT**  **Reverse** **CTG TCA TCC GTG CTT CCA GT** |
| ***ICAM-1*** | **Forward** **CCC TGC TCC GCT GCT G**  **Reverse** **GTT AGT CTC CAA CCC CAG GC** |
| ***P53*** | **Forward** **ATA TTC TGC CCA CCA CAG CG**  **Reverse** **AAG GGT GAA GTT GCC TTG GT** |
| ***GAPDH*** | **Forward** **CTC TCT GCT CCT CCC TGT TC**  **Reverse** **CGA CAT ACT CAG CAC CAG CA** |
| ***MMP-9*** | **Forward** **GCA TCT GTA TGG TCG TGG CT**  **Reverse CGT GCG GGC AAT AAG AAA GG** |
| ***IL-6*** | **Forward TCT GGT CTT CTG GAG TTC CGT**  **Reverse GGA TGG TCT TGG TCC TTA GCC** |
